# Dysregulated genes and miRNAs in the apoptosis pathway in colorectal cancer patients

**DOI:** 10.1007/s10495-018-1451-1

**Published:** 2018-03-07

**Authors:** Martha L. Slattery, Lila E. Mullany, Lori C. Sakoda, Roger K. Wolff, Wade S. Samowitz, Jennifer S. Herrick

**Affiliations:** 1 Department of Medicine, University of Utah, 383 Colorow, Salt Lake City, UT 84108 USA; 2 Division of Research, Kaiser Permanente Northern California, Oakland, CA USA; 3 Department of Pathology, University of Utah, Salt Lake City, UT USA

**Keywords:** Apoptosis, Colorectal cancer, miRNA, mRNA, *BIRC5*, *CTSS*

## Abstract

Apoptosis is genetically regulated and involves intrinsic and extrinsic pathways. We examined 133 genes within these pathways to identify whether they are expressed differently in colorectal carcinoma (CRC) and normal tissue (N = 217) and if they are associated with similar differential miRNA expression. Gene expression data (RNA-Seq) and miRNA expression data (Agilent Human miRNA Microarray V19.0) were generated. We focused on dysregulated genes with a fold change (FC) of > 1.50 or < 0.67, that were significant after adjustment for multiple comparisons. miRNA:mRNA seed-region matches were determined. Twenty-three genes were significantly downregulated (FC < 0.67) and 18 were significantly upregulated (FC > 1.50). Of these 41 genes, 11 were significantly associated with miRNA differential expression. *BIRC5* had the greatest number of miRNA associations (14) and the most miRNAs with a seed-region match (10). Four of these matches, miR-145-5p, miR-150-5p, miR-195-5p, and miR-650, had a negative beta coefficient. *CSF2RB* was associated with ten total miRNAs (five with a seed-region match, and one miRNA, miR-92a-3p, with a negative beta coefficient). Of the three miRNAs associated with *CTSS*, miR-20b-5p, and miR-501-3p, had a seed-region match and a negative beta coefficient between miRNA:mRNA pairs. Several miRNAs that were associated with dysregulated gene expression, seed-region matches, and negative beta coefficients also were associated with CRC-specific survival. Our data suggest that miRNAs could influence several apoptosis-related genes. *BIRC5, CTSS*, and *CSF2R* all had seed-region matches with miRNAs that would favor apoptosis. Our study identifies several miRNA associated with apoptosis-related genes, that if validated, could be important therapeutic targets.

**Electronic supplementary material:**

The online version of this article (10.1007/s10495-018-1451-1) contains supplementary material, which is available to authorized users.

## Introduction

Programmed cell death, or apoptosis, is genetically regulated [1]. Apoptosis is a homeostatic mechanism by which cell populations in tissues are maintained; it is a coordinated and energy-dependent process that involves activation of caspases (CASP), a group of cysteine proteases, followed by a cascade of events that ultimately end in cell death. Major caspases are classified into initiators of apoptosis (CASP 2, 8, 9, and 10), executioners of apoptosis (CASP 3, 6, and 7), and inflammatory caspases (CASP 1, 4, and 5). Activation of CASP8 triggers the execution phase of apoptosis; CASP3 is thought to be the most important executioner caspase. The two main linked pathways in apoptosis are the extrinsic or death receptor pathway and the intrinsic or mitochondrial pathway. The extrinsic signaling pathway includes tumor necrosis factor (TNF) receptor gene superfamily. The intrinsic signaling pathway involves mechanisms whereby signals can have either a positive or negative affect on apoptosis. Negative signals from the lack of specific growth factors, hormones, or cytokines can result in loss of apoptotic suppression and hence the activation of apoptosis. These changes also influence the mitochondrial membrane, activating the mitochondrial pathway. Control and regulation of these apoptotic mitochondrial events involves members of the Bcl-2 protein family. Members of the Bcl-2 protein family include anti-apoptotic proteins such as Bcl-2, Bcl-X, Bcl-XL, Bcl-XS, BclW and Bag as well as pro-apoptotic proteins such as Bcl-10, Bax, Bak, Bid, Bad, Bim, Bik, and Blk. These proteins are central in determining if apoptosis occurs. The IAP (inhibitor of apoptosis) family of proteins, which contains the Baculoviral IAP Repeat Containing (BIRC) genes, is an important regulator of apoptosis in that these proteins regulate both the extrinsic and intrinsic pathways.

MicroRNAs (miRNAs), small noncoding RNAs that bind to the 3′UTR of the protein coding mRNAs and inhibit their translation, may also be transcriptional targets. MiRNAs have been shown to be involved in many cellular processes, one of which is apoptosis. Several miRNAs, including miR-124 [2], miR-195 [3], miR-148a [4], miR-365 [5], miR-125b [6], miR-129 [7], miR-143 [8], and miR-203 [9], have been linked to apoptosis through regulation of genes such as *BCL2* and *PUMA*, a member of the Bcl-2 family, involved in pro-apoptosis [1, 4, 6, 8, 9].

Most studies of miRNAs and apoptosis have targeted specific miRNAs along with specific proteins or genes. In this study, we comprehensively evaluated all apoptosis genes identified in the Kyoto Encyclopedia of Genes and Genomes (KEGG) pathway to identify which genes are dysregulated in colorectal cancer (CRC). We compare dysregulated apoptosis genes with differentially expressed miRNAs to better define how miRNAs influence apoptosis. We utilized seed matches between associated mRNAs and miRNAs to determine if the associations are more likely to be direct, in that the binding of the miRNA to the mRNA directly influences the gene expression, or if the association is an indirect biological function in which the association between the miRNA and the mRNA is more likely to be from a feedback loop. A seed match would increase the likelihood that an identified miRNA:mRNA interaction was more likely to have a direct biological effect on expression given a higher propensity for binding. We further evaluated the impact of these dysregulated genes and miRNAs on CRC-specific survival.

## Methods

### Study participants

This study incorporates data from 217 individuals who participated in one of two population-based case-control studies of incident colon and rectal cancer patients in Utah or were members of Kaiser Permanente Northern California (KPNC). Participants were between 30 and 79 years of age, and self-reported being either non-Hispanic white, Hispanic, black, or Asian (rectal cancer study) [10, 11]. SEER (Surveillance, Epidemiology, and End Results) registries were used to verify cases as a first primary adenocarcinoma of the colon or rectum within the study-specific dates (October 1991 to September 1994 for the colon study or between May 1997 and May 2001 for the rectal study) [12]. SEER Registries in Northern California and Utah also provided information on tumor site, date of diagnosis, date of death or lost to follow-up, months of survival after diagnosis until either death or end of follow-up, and cause of death. The study was approved by the Institutional Review Boards at the University of Utah and at KPNC.

### RNA

Methods for RNA extraction, processing, and analysis have been described in detail [13, 14]. RNA was extracted from formalin-fixed paraffin embedded tissue from the initial biopsy or surgery, prior to chemotherapy treatment. For both mRNA and miRNA analysis, RNA was extracted from paired samples that consisted of the carcinoma tissue and adjacent normal mucosa as previously described [15]. Of the 245 individuals with sufficient tissue for analysis, 217 passed quality control (QC) were used in these analyses [16]. A more detailed description of the methods can be found in our previous work [17]. Total gene counts were calculated using gene coordinates obtained from http://genome.ucsc.edu. Genes that were not expressed in our RNA-Seq data or for which the expression was missing for the majority of samples were excluded from further analysis [17]. The Agilent Human miRNA Microarray V19.0 was used to generate miRNA data. QC parameters established by Agilent were implemented as previously described. Our previous assessment of miRNA expression repeatability was extremely high (r = 0.98) [12]. Further comparison of expression and fold change (FC) between paired tissue for the Agilent microarray with qPCR expression results showed 100% agreement when considering directionality of findings (i.e. expression either upregulated or downregulated in carcinoma tissue compared to normal) with FC being almost identical [18]. MiRNA expression was normalized by a scaling factor that was the median of the 75th percentile of all samples divided by the 75th percentile for each individual sample [19].

### KEGG-identified apoptosis genes

The KEGG pathway map program was used to identify a comprehensive list of genes within the apoptosis pathway (http://www.genome.jp/kegg-gin/show_pathway?hsa04210). We identified 138 genes (Supplemental Table S1) in this signaling pathway; 133 of these genes had sufficient expression in CRC tissue for statistical analysis.

### Statistical methods

We utilized a negative binomial mixed effects model in SAS, taking into account carcinoma/normal status as well as subject effect, to determine which genes had statistically significant difference in expression, either upregulated or downregulated, between paired carcinoma and normal mucosa. The log of the expression of all identified protein-coding genes in the negative binomial model (n = 17,461) was used to offset the overall exposure. The level of expression of each gene was calculated by dividing an individual’s total gene expression by the total expression of all protein-coding genes per million transcripts (RPMPCG or reads per million protein-coding genes). We calculated each individual’s FC between their carcinoma and normal mucosa expression. Our analysis with mRNAs and miRNAs focused on FCs of > 1.50 or < 0.67. The Benjamini and Hochberg [20] method was used to control the false discovery rate (FDR) using a value of < 0.05; this served as an adjustment for multiple comparisons. We considered overall CRC differential expression as well as differential expression specific for microsatellite unstable (MSI) and microsatellite stable (MSS) tumors to help determine genes that may have unique tumor phenotype associations.

We fit a least squares linear regression model to the RPMPCG differential expression levels and miRNA differential expression levels to determine mRNA:miRNA associations. We used the bootstrap method by creating a distribution of 10,000 F statistics derived by resampling the residuals from the null hypothesis model of no association between gene expression and miRNA expression using the boot package in R to generate p values. Our linear models were adjusted for age and sex. Multiplicity adjustments for gene mRNA/miRNA associations were made using the FDR by Benjamini and Hochberg [20].

Survival analyses were conducted using survival months calculated from diagnosis date to date of death or last follow-up, whichever occurred earlier. CRC-specific follow-up included deaths where the primary or secondary cause of death was listed as CRC. Individuals dying of other causes or who were lost to follow-up were censored at their time of death or date of last contact. We utilized the R package “survival” to calculate p values based upon 10,000 permutations of the likelihood ratio test from the Cox proportional hazards model adjusted for age at diagnosis, gender, and AJCC tumor stage at diagnosis. Reported Hazard Ratios (HR) are based on the difference between the 75th and 25th percentile of expression to help standardize differences in expression levels by miRNA. Assessment of miRNAs associated to apoptosis genes, utilized available data from 1134 colon cancer cases and 721 rectal cancer cases. This allowed us to analyze survival with colon and rectal cancer separately.

### Bioinformatics analysis

We assessed seed-region matches between mRNAs and miRNAs with FC of < 0.67 or > 1.50 that were statistically significant as described in our previous work [21]. We included seeds of six, seven, and eight nucleotides in length when determining seed-region matches. We believe that a seed match would increase the likelihood that an identified miRNA:mRNA interaction having a greater likelihood of a direct biological effect on expression since there are more likely to bind. Of particular importance are those mRNA:miRNA associations where the differential expression of one (either mRNA or miRNA) is inversely associated with the differential expression of the other. These associations are shown by a negative beta coefficient from the linear regression analysis. We used FASTA sequences generated from both GRCh37 and GRCh38 Homo sapiens, using UCSC Table Browser (https://genome.ucsc.edu/cgi-bin/hgTables) [22]. Detailed methods have been previously described [13, 14, 21].

## Results

The study population consisted of individuals who were predominately diagnosed with colon cancer (77.9%), were male (54.4%), were non-Hispanic white (74.2%), and had MSS tumors (86.6%) (Table 1). At the end of follow-up the majority of the population was alive (57.4%).

**Table 1:** Description of study population

	N	%
Site		
Colon	169	77.9
Rectal	48	22.1
Sex		
Male	118	54.4
Female	99	45.6
Age		
Mean (SD)	64.8	10.1
Race		
Non-Hispanic White	161	74.2
Hispanic	14	6.5
Non-Hispanic Black	8	3.7
Unknown	34	15.7
AJCC stage		
1	58	27.1
2	61	28.5
3	72	33.6
4	23	10.8
Tumor phenotype		
MSS	187	86.6
MSI	29	13.4
Vital status		
Dead	92	42.6
Alive	124	57.4

**Fig. 1:**
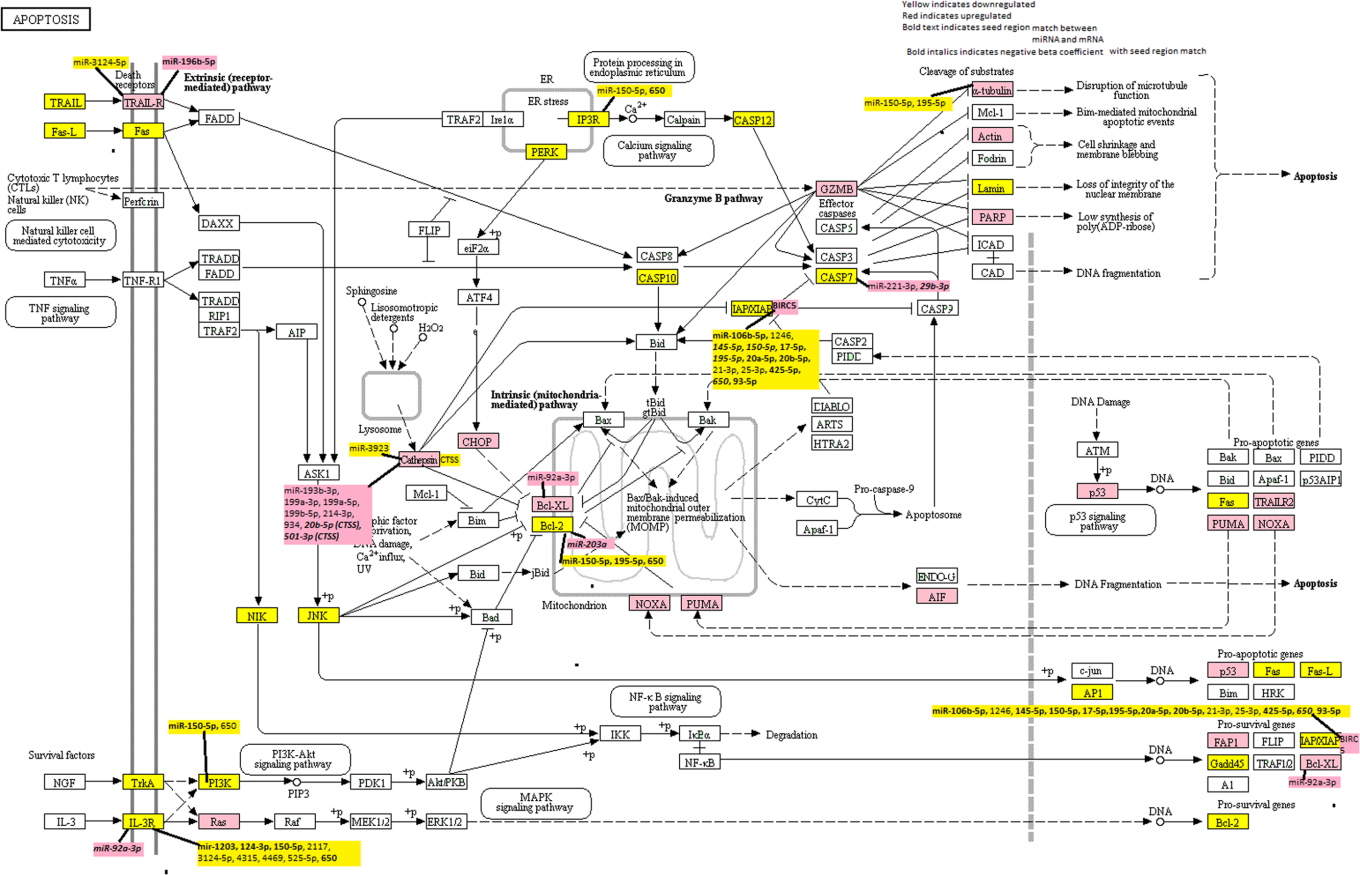
KEGG Apoptosis pathway with highlighted dysregulated mRNAs and miRNAs

Of the 133 genes analyzed, 23 were significantly downregulated with a FC < 0.67 and 18 were significantly upregulated with a FC > 1.5 (Table 2). Five genes, *SPTA1, MAPK10, TUBAL3, CSF2RB*, and *BCL2* all had a FC of < 0.4 and nine genes, *LMNB1, TUBA1C, PTPN13, LMNB2, BCL2L1, GZMB, BIRC5, PMAIP1*, and *CTSL2*, had a FC of > 2.0. Of the genes that were significantly differentially expressed in all CRC tumors combined, all but *TUBA3E*, were not downregulated in MSS tumors with a FC of < 0.67, and several genes (*NTRK1, FAS, CASP10, TUBA8, CTSS, FASLG, BLS2L11, LMNA, MAP3K14, BIRC3, and EIF2AK3*) were not downregulated in MSI tumors with a FC of < 0.67, although power was more limited when examined MSI-specific associations. However, two genes, *TNFSF10* and *EIF2AK3* (FCs 0.64) had a greater difference between carcinoma and normal mucosa in MSS-specific tumors (See Supplemental Table 2 for MSS-specific tumors), and three *CTSF*, had greater difference in MSI tumors (FC 0.44). Genes that were upregulated in all CRC combined were for the most part also upregulated to a similar degree in MSS-specific tumors, with the exception of *TNFRSF10B* (FC_all_ = 1.51, FC_MSS_ = 1.48) and *CTSH* (FC_all_ = 1.48, FC_MSS_ 1.59), which were slightly different FCs in MSS-specific tumors. As for genes that were downregulated, there were more differences in MSI-specific tumors than for genes that were upregulated. Four genes, *PARP4, AIFM1, CTSK*, and *GZMB*, were not upregulated > 1.50 for MSI tumors, while, *DDIT3, ACTG1, CTSL1, HRAS, and TNFRSF10D*, were more strongly upregulated in MSI-specific tumors (FCs 1.53, 1.55, 1.59, 1.92, and 1.503 respectively) (See Supplemental Table 3 for MSI-specific tumors). Figure 1 shows the up and downregulated genes in the KEGG Apoptosis Pathway. Genes that were specifically altered for only MSS and MSI carcinomas suggest unique tumor phenotype and disease pathways, whereas those seen for MSS, MSI and all CRC carcinomas suggest a broader application.

**Table 2:** Differentially expressed genes in the KEGG Apoptosis Pathway

Gene name	Tumor mean	Normal mean	Fold change	p value	Adjusted p value
*SPTA1*	0.73	2.32	0.32	1.73E−06	3.03E−06
*MAPK10*	9.26	28.10	0.33	8.93E−34	1.48E−32
*TUBAL3*	3.06	8.27	0.37	4.43E−16	1.44E−15
*CSF2RB*	27.82	73.57	0.38	7.06E−38	1.88E−36
*BCL2*	24.42	62.11	0.39	3.73E−39	1.24E−37
*FOS*	185.52	453.52	0.41	9.65E−31	1.07E−29
*CASP12*	0.42	0.94	0.45	7.00E−04	1.02E−03
*NTRK1*	1.10	2.43	0.45	3.24E−07	5.98E−07
*ITPR1*	57.21	109.94	0.52	5.07E−36	9.63E−35
*FAS*	28.16	50.95	0.55	2.75E−24	1.66E−23
*CASP10*	65.13	111.98	0.58	5.20E−32	6.92E−31
*PIK3CD*	27.33	46.48	0.59	9.96E−22	4.42E−21
*TUBA8*	3.28	5.46	0.60	8.69E−09	1.75E−08
*IL3RA*	2.21	3.59	0.62	2.82E−03	3.95E−03
*CASP7*	59.81	96.30	0.62	1.52E−24	1.01E−23
*TUBA3E*	0.42	0.68	0.62	9.17E−03	1.22E−02
*CTSS*	113.76	180.97	0.63	1.46E−23	7.76E−23
*FASLG*	1.07	1.70	0.63	4.96E−03	6.74E−03
*BCL2L11*	56.19	88.47	0.64	1.23E−23	6.82E−23
*LMNA*	245.65	386.57	0.64	3.99E−20	1.66E−19
*MAP3K14*	33.86	51.73	0.65	1.46E−24	1.01E−23
*BIRC3*	79.53	120.36	0.66	1.83E−14	5.17E−14
*EIF2AK3*	52.04	78.19	0.67	3.08E−23	1.57E−22
*MAPK3*	62.34	92.73	0.67	9.12E−19	3.37E−18
*TNFSF10*	48.33	71.22	0.68	1.22E−12	3.07E−12
*CFLAR*	203.49	290.53	0.70	2.89E−29	2.96E−28
*CTSW*	2.82	3.99	0.71	4.76E−04	7.11E−04
*RIPK1*	49.35	69.68	0.71	1.19E−19	4.53E−19
*ERN1*	27.13	38.17	0.71	4.23E−11	9.87E−11
*BAD*	13.13	18.42	0.71	1.38E−11	3.28E−11
*CAPN2*	244.01	340.39	0.72	1.09E−22	5.38E−22
*CASP9*	13.05	17.74	0.74	1.52E−08	3.01E−08
*TP53AIP1*	0.61	0.81	0.75	1.59E−01	1.87E−01
*TNFRSF1A*	90.65	118.74	0.76	4.32E−20	1.74E−19
*ATM*	239.32	312.70	0.77	4.82E−16	1.52E−15
*DAB2IP*	144.00	186.15	0.77	1.71E−14	4.95E−14
*MCL1*	615.27	794.46	0.77	3.54E−14	9.82E−14
*CYCS*	95.12	122.36	0.78	1.03E−10	2.35E−10
*NFKBIA*	58.77	75.03	0.78	1.13E−07	2.17E−07
*AKT3*	41.45	52.56	0.79	2.51E−05	4.28E−05
*CTSF*	7.12	8.89	0.80	1.10E−02	1.44E−02
*BAK1*	20.05	24.24	0.83	2.77E−05	4.60E−05
*CTSD*	270.28	324.99	0.83	4.20E−07	7.66E−07
*BIRC2*	81.92	98.12	0.83	6.71E−10	1.44E−09
*TRADD*	25.11	28.92	0.87	2.47E−03	3.53E−03
*ITPR2*	221.72	255.25	0.87	3.28E−03	4.55E−03
*GADD45G*	2.79	3.18	0.88	2.07E−01	2.39E−01
*RAF1*	131.53	147.29	0.89	5.78E−07	1.04E−06
*TRAF1*	46.52	51.87	0.90	1.55E−02	1.98E−02
*KRAS*	107.64	117.88	0.91	1.65E−02	2.09E−02
*MAP2K2*	69.97	75.76	0.92	1.47E−02	1.90E−02
*NFKB1*	77.63	83.98	0.92	7.65E−03	1.03E−02
*MAP3K5*	68.22	73.59	0.93	3.82E−02	4.71E−02
*PIDD*	28.99	31.19	0.93	1.08E−01	1.30E−01
*PARP3*	18.95	20.17	0.94	2.44E−01	2.78E−01
*IKBKB*	137.36	145.80	0.94	6.63E−02	8.08E−02
*APAF1*	85.50	90.73	0.94	7.64E−02	9.24E−02
*PARP2*	20.40	21.49	0.95	3.35E−01	3.68E−01
*SEPT4*	6.07	6.38	0.95	5.52E−01	5.78E−01
*TNF*	1.95	2.04	0.96	7.52E−01	7.58E−01
*CASP3*	42.94	44.90	0.96	3.39E−01	3.69E−01
*CTSC*	83.85	87.45	0.96	2.63E−01	2.93E−01
*TNFRSF10D*	21.48	22.33	0.96	5.20E−01	5.49E−01
*TUBA3D*	2.86	2.96	0.97	7.43E−01	7.55E−01
*PRF1*	5.19	5.24	0.99	9.15E−01	9.15E−01
*AKT1*	153.83	152.34	1.01	7.08E−01	7.24E−01
*JUN*	214.48	211.03	1.02	7.02E−01	7.24E−01
*MAPK8*	62.01	60.98	1.02	6.19E−01	6.43E−01
*MAPK1*	183.50	179.79	1.02	3.53E−01	3.78E−01
*MAPK9*	65.92	64.12	1.03	4.12E−01	4.39E−01
*PIK3R1*	154.42	149.09	1.04	3.00E−01	3.32E−01
*PIK3R3*	35.88	33.87	1.06	2.55E−01	2.87E−01
*CASP6*	22.61	21.28	1.06	2.10E−01	2.41E−01
*GADD45B*	12.90	12.05	1.07	3.43E−01	3.71E−01
*PDPK1*	100.97	93.03	1.09	3.80E−03	5.21E−03
*IKBKG*	6.91	6.20	1.11	2.04E−01	2.38E−01
*PIK3CB*	90.89	81.36	1.12	5.56E−04	8.21E−04
*RELA*	82.20	73.32	1.12	5.56E−06	9.60E−06
*CTSZ*	123.07	109.77	1.12	2.59E−03	3.66E−03
*ITPR3*	420.93	374.92	1.12	2.74E−05	4.60E−05
*DIABLO*	39.85	35.12	1.13	6.78E−05	1.07E−04
*CAPN1*	187.82	164.50	1.14	1.79E−07	3.41E−07
*ENDOG*	12.68	11.09	1.14	2.72E−02	3.41E−02
*GADD45A*	13.07	11.37	1.15	3.07E−02	3.81E−02
*AKT2*	156.81	135.87	1.15	2.66E−07	4.99E−07
*SPTAN1*	543.11	467.35	1.16	3.33E−10	7.38E−10
*MAP2K1*	32.75	28.06	1.17	4.28E−04	6.55E−04
*CTSL1*	20.11	17.18	1.17	1.18E−02	1.53E−02
*CHUK*	44.48	37.81	1.18	4.50E−04	6.80E−04
*HTRA2*	19.32	16.19	1.19	5.92E−05	9.61E−05
*XIAP*	187.63	156.09	1.20	8.16E−13	2.13E−12
*PIK3CA*	67.11	55.23	1.22	6.77E−07	1.20E−06
*FADD*	14.64	12.03	1.22	7.19E−04	1.04E−03
*BAX*	36.23	29.75	1.22	1.59E−04	2.49E−04
*CASP8*	70.64	57.92	1.22	3.90E−09	8.11E−09
*DFFB*	16.02	13.07	1.23	1.87E−04	2.89E−04
*ATF4*	130.84	105.67	1.24	8.92E−13	2.28E−12
*BCL2A1*	2.01	1.61	1.25	1.59E−01	1.87E−01
*DAXX*	35.66	28.09	1.27	3.63E−10	7.92E−10
*BID*	35.72	27.95	1.28	2.19E−09	4.63E−09
*TNFRSF10A*	37.99	28.64	1.33	2.17E−08	4.25E−08
*PIK3R2*	72.83	54.55	1.34	1.32E−18	4.73E−18
*DDIT3*	11.07	8.29	1.34	3.01E−05	4.93E−05
*ACTB*	2123.22	1584.09	1.34	3.58E−28	3.18E−27
*NRAS*	104.64	76.74	1.36	3.26E−15	9.64E−15
*TRAF2*	40.20	29.46	1.36	8.02E−13	2.13E−12
*PARP1*	109.63	79.73	1.37	5.66E−16	1.75E−15
*EIF2S1*	70.00	50.27	1.39	6.06E−16	1.83E−15
*DFFA*	86.27	61.10	1.41	2.64E−24	1.66E−23
*TUBA1A*	27.86	19.65	1.42	8.44E−09	1.73E−08
*CASP2*	124.24	87.36	1.42	9.53E−25	7.04E−24
*CTSB*	513.97	360.55	1.43	2.50E−22	1.19E−21
*ACTG1*	1209.84	823.20	1.47	6.71E−29	6.38E−28
*CTSH*	76.75	51.92	1.48	5.16E−14	1.40E−13
*HRAS*	18.52	12.37	1.50	3.52E−12	8.66E−12
*PARP4*	340.90	226.33	1.51	2.95E−26	2.45E−25
*TNFRSF10B*	135.53	89.65	1.51	3.45E−21	1.48E−20
*AIFM1*	41.19	27.11	1.52	4.56E−17	1.55E−16
*CTSK*	48.58	31.12	1.56	8.29E−12	2.00E−11
*TNFRSF10C*	3.54	2.10	1.69	6.25E−05	1.00E−04
*TUBA4A*	33.42	19.75	1.69	3.16E−22	1.45E−21
*TP53*	105.07	59.63	1.76	3.25E−24	1.88E−23
*BBC3*	20.58	11.49	1.79	2.02E−18	7.06E−18
*TUBA1B*	138.84	76.60	1.81	3.01E−31	3.64E−30
*LMNB1*	89.36	43.88	2.04	1.01E−36	2.23E−35
*TUBA1C*	69.89	33.41	2.09	1.39E−32	2.05E−31
*PTPN13*	69.58	32.80	2.12	4.54E−20	1.78E−19
*LMNB2*	150.48	68.95	2.18	1.08E−45	7.20E−44
*BCL2L1*	144.28	64.05	2.25	1.06E−56	1.41E−54
*GZMB*	4.21	1.49	2.83	2.11E−10	4.77E−10
*BIRC5*	35.63	11.31	3.15	1.67E−39	7.41E−38
*PMAIP1*	11.00	3.31	3.32	6.06E−26	4.74E−25
*CTSL2*	4.38	1.21	3.64	8.07E−17	2.68E−16

Of the 41 genes with a FC of < 0.67 or > 1.50 analyzed with miRNA differential expression, 11 were significantly associated after adjustment for multiple comparisons (Table 3). There were a total of 48 miRNA:mRNA associations with *BIRC5* having the greatest number of miRNA associations. Ten of the 14 miRNAs associated with *BIRC5* has a seed-region match, and four of these matches, miR-145-5p, miR-150-5p, miR-195-5p, and miR-650, had a negative beta coefficient. *CSF2RB* was associated with ten miRNAs, five of which had a seed-region match, and one miRNA, miR-92a-3p, had a negative beta coefficient. Two miRNAs, miR-20b-5p, and miR-501-3p associated with *CTSS* had both a seed-region match and a negative beta coefficient between the differentially expressed gene and the differentially expressed miRNA. Other genes and miRNA pairs that had both a seed-region match and a negative beta coefficient were *TNFRSF10* with miR-196b-5p, *CASP7* with miR-29b-3p, and *BCL2* with miR-203a. Several miRNAs were associated with multiple genes, including miR-150-5p with six genes (*BIRC5, CSF2RF, TUBA1B, IRPR1, PIK3CD*, and *BCL2*), miR-650 with five genes (*BIRC5, CSF2RB, ITPR1, PIK3CD*, and *BCL2*), miR-195-5p with three genes (*BIRC5, TUB1B*, and *BCL2*), and miR-20b-5p and miR-3124-5p each with two genes (miR-20b-5p with *BIRC5* and *CTSS* and miR-3124-5p with *TNFRSF10B* and *CSF2RB*).

**Table 3:** Differentially expressed genes in the KEGG apoptosis pathway associated with miRNA differential expression

Gene name	Tumor mean	Normal mean	Fold change	miRNA	Tumor mean	Normal mean	Fold change	Beta	Raw p value	FDR p value
*BIRC5*	35.63	11.31	3.15	**hsa-miR-106b-5p** ^**a**^	15.90	5.19	3.06	0.23	0.0007	0.0285
				hsa-miR-1246	629.21	412.81	1.52	0.25	0.0006	0.0257
				**hsa-miR-145-5p**	132.97	223.14	0.60	**− 0.32**	< .0001	0.0102
				**hsa-miR-150-5p**	14.90	39.17	0.38	**− 0.24**	0.0003	0.0174
				**hsa-miR-17-5p**	61.04	16.38	3.73	0.28	< .0001	0.0102
				**hsa-miR-195-5p**	3.59	12.18	0.29	**− 0.25**	0.0005	0.0226
				hsa-miR-19b-3p	29.80	10.42	2.86	0.28	< .0001	0.0102
				**hsa-miR-20a-5p**	70.78	17.61	4.02	0.25	0.0004	0.0192
				**hsa-miR-20b-5p**	17.65	3.30	5.35	0.28	< .0001	0.0102
				hsa-miR-21-3p	22.68	9.89	2.29	0.28	0.0002	0.0136
				hsa-miR-25-3p	30.05	12.78	2.35	0.31	< .0001	0.0102
				**hsa-miR-425-5p**	11.76	6.97	1.69	0.25	0.0003	0.0174
				**hsa-miR-650**	4.51	16.60	0.27	**− 0.26**	0.0002	0.0136
				**hsa-miR-93-5p**	41.72	15.20	2.74	0.31	< .0001	0.0102
*CSF2RB*	27.82	73.57	0.38	**hsa-miR-1203**	1.76	2.83	0.62	0.23	0.0011	0.0497
				**hsa-miR-124-3p**	0.90	2.40	0.38	0.25	0.0005	0.037
				**hsa-miR-150-5p**	14.90	39.17	0.38	0.30	< .0001	0.0102
				hsa-miR-2117	1.50	4.09	0.37	0.28	0.0003	0.0244
				hsa-miR-3124-5p	1.37	2.27	0.60	0.28	< .0001	0.0102
				hsa-miR-4315	0.21	2.62	0.08	0.23	0.0008	0.0497
				hsa-miR-4469	1.11	2.41	0.46	0.22	0.0011	0.0497
				hsa-miR-525-5p	1.56	2.53	0.62	0.22	0.001	0.0497
				**hsa-miR-650**	4.51	16.60	0.27	0.37	< .0001	0.0102
				**hsa-miR-92a-3p**	121.60	41.18	2.95	**− 0.24**	0.0009	0.0497
*TNFRSF10B*	135.53	89.65	1.51	**hsa-miR-196b-5p**	17.89	5.53	3.24	**− 0.25**	0.0003	0.0488
				hsa-miR-3124-5p	1.37	2.27	0.60	− 0.26	0.0003	0.0488
*TUBA1B*	138.84	76.60	1.81	hsa-miR-150-5p	14.90	39.17	0.38	− 0.25	< .0001	0.0163
				hsa-miR-195-5p	3.59	12.18	0.29	− 0.28	0.0002	0.0181
*CTSK*	48.58	31.12	1.56	hsa-miR-193b-3p	9.12	5.42	1.68	0.26	0.0003	0.0271
				hsa-miR-199a-3p	44.83	22.53	1.99	0.35	< .0001	0.0102
				hsa-miR-199a-5p	20.18	9.28	2.17	0.33	< .0001	0.0102
				hsa-miR-199b-5p	4.69	1.53	3.07	0.34	< .0001	0.0102
				hsa-miR-214-3p	13.24	6.13	2.16	0.38	< .0001	0.0102
				hsa-miR-934	4.36	0.94	4.66	0.43	< .0001	0.0102
*ITPR1*	57.21	109.94	0.52	hsa-miR-150-5p	14.90	39.17	0.38	0.34	< .0001	0.0271
				**hsa-miR-650**	4.51	16.60	0.27	0.33	< .0001	0.0271
*CTSS*	113.76	180.97	0.63	**hsa-miR-20b-5p**	17.65	3.30	5.35	**− 0.25**	0.0003	0.0488
				hsa-miR-3923	0.29	1.66	0.18	− 0.28	0.0002	0.0488
				**hsa-miR-501-3p**	7.07	2.95	2.39	**− 0.29**	< .0001	0.0407
*CASP7*	59.81	96.30	0.62	hsa-miR-221-3p	13.53	4.12	3.28	− 0.32	< .0001	0.0407
				**hsa-miR-29b-3p**	24.31	9.83	2.47	**− 0.30**	< .0001	0.0407
*BCL2L1*	144.28	64.05	2.25	hsa-miR-92a-3p	121.60	41.18	2.95	0.34	< .0001	0.0407
*PIK3CD*	27.33	46.48	0.59	**hsa-miR-150-5p**	14.90	39.17	0.38	0.35	< .0001	0.0271
				hsa-miR-650	4.51	16.60	0.27	0.31	< .0001	0.0271
*BCL2*	24.42	62.11	0.39	**hsa-miR-150-5p**	14.90	39.17	0.38	0.34	< .0001	0.0203
				**hsa-miR-195-5p**	3.59	12.18	0.29	0.24	0.0003	0.0488
				**hsa-miR-203a**	12.52	3.70	3.38	**− 0.27**	< .0001	0.0203
				**hsa-miR-650**	4.51	16.60	0.27	0.38	< .0001	0.0203

^a^Bold text indicates seed-region match between miRNA and mRNA

There were no significant associations between differentially expressed mRNAs and survival. However, evaluation of those miRNAs associated with differentially expressed genes with survival, showed that 11 genes were associated with survival with both significant raw p values and Q-values less than 0.05 (Table 4). Increased miRNA expression of miR-124-3p, miR-145-3p, miR-193b-3p, and miR-934 in carcinomas was associated with worse survival while increased expression of other miRNAs (i.e. miR-17-5p, miR-19b-3p, miR-20a-5p, miR-20b-5p, miR-425-5p, miR-92a-3p, and miR-93-5p) in carcinoma tissue improved survival. Four miRNAs were associated with survival after being diagnosed with colon cancer, miR-124-3p, miR-145-5p, miR-193b-3p, and miR-934, although none of these associations remained significant after adjustment for multiple comparisons. However, 16 miRNAs were associated with survival after a diagnosis with rectal cancer after adjustment for multiple comparisons. Ten of these miRNAs had seed-region matches and six of these miRNAs, miR-150-5p, miR-196b-5p, miR-203a, miR-20b-5p, miR-501-3p, and miR-92a-3p, had negative beta coefficients between differentially expressed miRNA and mRNA, suggesting a greater likelihood for direct binding that would alter the gene expression. In all instances, having greater expression of miRNA in the carcinoma than in the normal mucosa resulted in improved survival.

**Table 4:** Associations between miRNAs associated with KEGG apoptosis pathway and colorectal cancer survival

miRNA	Q1	Q3	HR^a^	95% CI	p value	Q value	FDR adjusted p
All colorectal cancer							
**hsa-miR-124-3p**^b^	− 0.36	0.00	1.04	1.00–1.07	0.04	0.04	0.14
***hsa-miR-145-5p***	− 1.94	− 0.15	1.13	1.01–1.26	0.03	0.03	0.14
***hsa-miR-17-5p***	0.95	2.40	0.91	0.84–0.98	0.02	0.03	0.14
hsa-miR-193b-3p	− 0.16	1.45	1.10	1.01–1.20	0.03	0.03	0.14
hsa-miR-19b-3p	0.61	2.35	0.91	0.84–0.99	0.02	0.03	0.14
**hsa-miR-20a-5p**	1.02	2.61	0.91	0.84–0.98	0.02	0.03	0.14
***hsa-miR-20b-5p***	0.96	3.11	0.83	0.75–0.91	0.0002	0.03	0.01
**hsa-miR-425-5p**	− 0.10	1.48	0.92	0.85–0.998	0.04	0.04	0.14
hsa-miR-92a-3p	0.63	1.87	0.91	0.83–0.99	0.03	0.03	0.14
hsa-miR-934	0.50	2.29	1.13	1.01–1.27	0.03	0.03	0.14
**hsa-miR-93-5p**	0.71	1.99	0.93	0.86–0.996	0.04	0.04	0.14
Colon cancer							
**hsa-miR-124-3p**	− 0.36	0.00	1.05	1.01–1.10	0.02	NA^c^	0.21
***hsa-miR-145-5p***	− 1.88	− 0.03	1.18	1.03–1.36	0.02	NA	0.21
hsa-miR-193b-3p	− 0.19	1.66	1.19	1.05–1.34	0.004	NA	0.13
Rectal cancer							
hsa-miR-106b-5p	0.71	2.64	0.80	0.68–0.94	0.01	0.06	0.03
hsa-miR-1203	− 1.44	0.00	0.82	0.69–0.96	0.02	0.06	0.04
***hsa-miR-150-5p***	− 2.49	− 0.73	0.82	0.70–0.96	0.01	0.06	0.04
***hsa-miR-17-5p***	1.13	2.43	0.80	0.70–0.91	0.00	0.06	0.01
***hsa-miR-196b-5p***	0.00	3.21	0.73	0.59–0.89	0.00	0.06	0.01
hsa-miR-19b-3p	0.82	2.39	0.80	0.70–0.91	0.00	0.06	0.01
***hsa-miR-203a***	0.00	2.76	0.82	0.68–0.99	0.04	0.07	0.08
**hsa-miR-20a-5p**	1.20	2.70	0.79	0.69–0.91	0.00	0.06	0.01
***hsa-miR-20b-5p***	1.24	3.30	0.67	0.57–0.79	0.00	0.06	0.00
hsa-miR-21-3p	0.50	1.62	0.85	0.75–0.97	0.02	0.06	0.04
hsa-miR-221-3p	0.63	2.57	0.81	0.69–0.94	0.01	0.06	0.03
hsa-miR-25-3p	0.63	1.84	0.86	0.76–0.98	0.02	0.06	0.05
**hsa-miR-425-5p**	0.00	1.54	0.84	0.74–0.95	0.01	0.06	0.03
***hsa-miR-501-3p***	0.57	1.73	0.81	0.69–0.94	0.01	0.06	0.03
hsa-miR-525-5p	− 0.02	0.62	0.91	0.84–0.98	0.01	0.06	0.04
***hsa-miR-92a-3p***	0.78	1.86	0.78	0.67–0.91	0.00	0.06	0.01
**hsa-miR-93-5p**	0.88	1.98	0.85	0.76–0.96	0.01	0.06	0.03

^a^Hazard ratios (HR) and 95% confidence intervals (CI) adjusted for age, sex, and AJCC stage; HR calculated as the risk associated with the difference between Q1 and Q3

^b^Bold text indicates seed-region match with mRNA; italics indicates negative beta coefficient between differential expression of mRNA and miRNA for those with a seed-region match

^c^Q-value not calculated for colon-cancer specific mortality because the estimated number of true null hypothesis is zero

## Discussion

Our data suggest that miRNAs are involved in apoptosis at several key junctions that involve both the extrinsic pathway, given associations with TRAIL-R (*TNFRSF10B*) and *IP3R, BIRC5*, and *CASP7*, as well as with the intrinsic pathway via associations with Cathepsin and members of the Bcl-2 family and inflammation via CSF2RB (IL3RB) and PI3K. Several miRNAs associated with this pathway also were associated with survival. Our findings identify key points within the pathway that may be suitable for further research and potential targets for therapeutic intervention.

By utilizing seed-region matches we were able to identify miRNAs that were more likely to have a direct biological effect on the mRNA that could in turn influence the apoptosis pathway. MiRNA binding with the 3′UTR of the mRNA increases the likelihood of mRNA degradation; this is especially relevant when increases in differential expression of miRNA results in decreases in differential expression of mRNA. This suggests that the miRNA is directly influencing the mRNA expression and may represent an area in the pathway where miRNAs can directly exert influence on the apoptotic process. Indirect effects are most likely operating feed-back and feed-forward loops [23–25]. In feed-back loops, regulators such as miRNAs and transcription factors (TFs) can have either the same effect (repression of expression) or opposite effects where the TF enhances the mRNA [24]. In feed-forward loops, TF regulates the miRNA as well as a target gene (TG), which is in turn also regulated by the miRNA. In this instance, the miRNA may regulate the TG directly, through seed region binding leading to mRNA degradation or translational repression, or indirectly, through repression of the TF that is influencing transcription of the same TG. Regulatory networks involving miRNAs are believed to be prevalent mechanisms for modulating gene expression [24]. While indirect effects are most likely important in the apoptosis pathway, the direct binding between miRNA and mRNA that results in inverse expression associations have a greater potential as a target of future research and is the focus of the discussion of our results.

Cathepsins are a family of “lysosomal proteolytic enzymes” [26] that are released from lysosomes into the cytoplasm, triggering apoptosis. They are involved in both the intrinsic pathway, regulating the release of pro-apoptotic factors from the mitochondria, but also are involved in the extrinsic pathway of apoptosis given the ability to block inhibitors of apoptosis (IAP). In our study both *CTSK* (cathepsin K) and *CTSS* (cathepsin S) were associated with miRNAs, however, only *CTSS* was directly associated given the likely binding of miR-20b-5p and miR-501-3p that could result in a decreased expression of *CTSS* in the presence of increased differential expression of these miRNAs. *CTSS* is a cysteine cathepsin that has been linked previously to tumorigenesis and to response to chemotherapy in CRC patients [27]. Studies have suggested that cathepsin S promotes tumor invasion through extracellular matrix degradation that release matrix—derived growth factors that drive angiogenesis [28]. Cathepsin S can block members of the Bcl-2 family as well as IAPs. Given that *CTSS* was down-regulated, two components of the pathway could be affected. *BCL2L1*, an apoptosis inhibitor, was possibly up-regulated since *CTSS* could not inhibit it. Another member of the Bcl-2 family, anti-apoptotic *BCL2*, was down-regulated. *BCL2* was directly associated through seed-region match to miR-203a, which could also result in decreased expression of *BCL2*, which would activate the intrinsic apoptosis pathway. *BLC2* has been examined with several targeted miRNAs, including miR-491, miR-143, miR-148a, miR-365, miR-1915, miR-204, and miR-125b [4–6, 8, 29–31]. We did not see direct associations with any of these genes, however we could have missed associations since we only examined gene expression data and protein activity could have been altered. We did note that *BCL2* had seed-region matches with all of these miRNAs except miR-491, suggesting that associations likely exist but were not detected in this study, possibly because of an effect on protein level rather than on mRNA expression. Also, given our study design and adjustment for multiple comparisons we could have not detected significant associations in our broader study. Likewise, our uniquely identified associations with *BCL2* and miRNAs most likely reflects that they have not being previously examined, as much of the literature is based on targeted miRNAs rather than all miRNAs commonly expressed in colorectal tissue [32]. We believe that these miRNAs may be key targets in therapeutics given the apparent role in regulating apoptosis. Our pathway approach to examining miRNAs has hopefully provided insight into unique role of miRNAs as there relate to the carcinogenic process.

In addition to altering the intrinsic pathway, *CTSS* has a role in blocking IAPs. In our data *BIRC5*, which encodes for the protein survivin, was one of the most up-regulated genes in the apoptosis pathway. *BIRC5* inhibits caspase activity and overexpression of *BIRC5* leads to resistance to apoptosis [33]. Survivin blocks apoptosis by binding to caspases, such as *CASP7*, an executioner of the apoptosis process that has been explored as a target for cancer therapeutics [34]. In our data, *BIRC5* was the gene that had the most seed-region matches with miRNAs, four of which were down-regulated when *BIRC5* was up-regulated. The miRNAs that appear to directly target *BIRC5* are miR-145-5p, miR-150-5p, miR-195-5p, and miR-650; both miR-145-5p and miR-150-5p were associated with better survival after diagnosed with CRC overall or with rectal cancer specifically when upregulated in CRC tumor tissue. Taken together, these results suggest that decreases in the expression of these miRNAs allowed greater expression of *BIRC5* and down-regulation of *CTSS*, resulting in less inhibition of *BIRC5* and possibly resistance to apoptosis. When *BIRC5* was upregulated, *CASP7* was likely inhibited, as indicated by decreased expression in tumors, as was the predicted effect of a decrease in apoptosis. *CASP7* also had a direct biological association with miR-29b-3p, supported by a seed region match and a negative beta coefficient for this association, which would suggest further decreases *CASP7* expression. Others have identified miR-203 as being associated with survivin [35], although we did not observe this association we did note that there was a seed-region match between miR-203b-5p and *BIRC5*. The miRNAs identified in our study, miR-145-5p, miR-150-5p, miR-195-5p, miR-650, and miR-29b-3p, may serve as reasonable therapeutic targets given their likely role in influencing activity of *BIRC5* and *CASP7*, two important elements in the apoptosis pathway.

A third area of importance in the apoptosis pathway is CSF2RB (Colony Stimulating Factor 2 Beta Common Subset B), or IL-3R, which is a type 1 cytokine receptor. *CSF2RB* was down-regulated (FC 0.38) and associated with 10 miRNAs; miR-92a-3p was up-regulated as *CSF2RB* was down-regulated and there was a seed-region match between the gene and miRNA. Down-regulation of *CSF2RB* could lead to down-regulation of PI3K genes (*PIK3CD* was down-regulated in our data), and alter the MAPK and NFκB-signaling pathways that could ultimately lead to altered expression of genes needed for regulating apoptosis of certain inflammatory cells.

While we did not observe an association between dysregulated genes in the apoptosis pathway and survival, it should be kept in mind that we had limited statistical power to evaluate associations. Power was further limited to evaluate site-specific associations with dysregulated genes. However, evaluation of miRNAs with survival was feasible because for that component of the analysis we had a much larger sample of colon cancer cases and rectal cancer cases with paired miRNA expression data. Our approach was to thus evaluate miRNAs that were associated with mRNAs in the pathway and determine their association with survival. We observed that several miRNAs were associated with CRC-specific survival for colon and rectal cases combined and that the strongest associations were between miRNAs and survival after being diagnosed with rectal cancer. Several of the miRNAs associated with survival appeared to be directly biologically associated with mRNAs, in that an increase in the differential expression of the miRNA reduced the differential mRNA expression. Several of these miRNAs had direct associations with *BIRC5*, including miR-145-5p and miR-17-5p for all CRC and miR-150-5p and miR-196b-5p for rectal cancer specifically. MiR-20b-5p and miR-501-3p were associated with *CTSS*, miR-92a-5p was associated with *CSF2RB*, and miR-203a was inversely associated with *BCL2*. These findings suggest that miRNA binding, or lack of binding, results in an increase (i.e. *BIRC5*) in anti-apoptotic gene expression or a decrease (i.e. *CSF2RB, CTSS*, and *BCL2*) in pro-apoptotic gene expression, which could result in decreased apoptosis, which could favor tumorigenesis and could ultimately influence patient prognosis.

Our sample size is one of the largest available that contains individuals with both carcinoma and normal mucosa expression data. We acknowledge that normal colonic mucosa, while not being entirely normal since it too may have undergone changes from health colonic mucosa, is the closest colonic tissue available for a matched-paired analysis. The normal colonic mucosa utilized was taken from the same colonic site as the tumor; this prevented differences in expression between carcinoma and normal mucosa, from being the result of tumor location. As we stated previously, there are differences in our identified associations from those reported in the literature; this could stem in part from our analysis based on paired samples. A study limitation is our sample size of mRNA data, which would greatly impact our ability to identify associated between dysregulated genes and survival. In interpreting the results of this research it should be acknowledged that FC cut point for assessment of meaningful changes is arbitrary. Utilization of FC cutpoints of < 0.67 or > 1.50 enabled us to focus on changes that might be more biologically meaningful. Small FCs, say of 0.9 or 1.1, could stem from measurement differences, sample processing, or other factors that lead to small amounts of expression that may not be meaningful. Using these criteria, we examined what we believed to be the most meaningful changes when looking at mRNA:miRNA associations and therefore could have missed other significant associations. We exclusively used the KEGG pathway database to identify apoptosis pathway genes; genes not identified in KEGG but may influence apoptosis as well as influence miRNA expression were not analyzed. Another study limitation is availability of only gene expression data. Since miRNAs may have their impact post-transcriptionally, protein expression data could provide additional insight into associations. However, it should be acknowledged that much of the current information on miRNA target genes comes from gene expression data and associations observed may have important biological meaning [32, 36]. Studies have been conducted that suggest that even though miRNAs have their impact at the post-transcriptional level, they still commonly alter gene expression [37, 38]. While conducting more detailed functionality assessment of the interaction between mRNA and miRNA is needed, it is beyond the scope of this study.

In conclusion, our data suggest several key areas in the apoptosis pathway where miRNAs may alter expression of genes such as *BIRC5, CASP7, CTSS, CSF2RB*, and *BCL2*, that in turn influences apoptosis and tumorigenesis. The results from this study provide important targets for follow-up in laboratory-based functionality studies that could lead to new cancer therapeutics. We encourage others to replicate these findings and validate their feasibility as therapeutic targets.

## Electronic supplementary material

Below is the link to the electronic supplementary material.

Supplementary material 1 (DOCX 38 KB)
