# Supplementary material for: Dysregulated genes and miRNAs in the apoptosis pathway in colorectal cancer patients
Source: Apoptosis. 2018 Mar 7;23(3):237–50. doi: 10.1007/s10495-018-1451-1 (PMC5856858; doi:10.1007/s10495-018-1451-1)
Supplement: Supplementary file 1 — Supplementary material 1 (DOCX 38 KB) [file 10495_2018_1451_MOESM1_ESM.docx]

| Supplemental Table 1. Genes indentified in the KEGG Apoptosis Pathway | | | |
| --- | --- | --- | --- |
| Gene ID | Gene Name | Description |  |
| 60 | *ACTB* | actin beta [KO:K05692] | |
| 71 | *ACTG1* | actin gamma 1 [KO:K05692] | |
| 9131 | *AIFM1* | apoptosis inducing factor mitochondria associated 1 [KO:K04727] [EC:1.-.-.-] | |
| 207 | *AKT1* | AKT serine/threonine kinase 1 [KO:K04456] [EC:2.7.11.1] | |
| 208 | *AKT2* | AKT serine/threonine kinase 2 [KO:K04456] [EC:2.7.11.1] | |
| 10000 | *AKT3* | AKT serine/threonine kinase 3 [KO:K04456] [EC:2.7.11.1] | |
| 317 | *APAF1* | apoptotic peptidase activating factor 1 [KO:K02084] | |
| 468 | *ATF4* | activating transcription factor 4 [KO:K04374] | |
| 472 | *ATM* | ATM serine/threonine kinase [KO:K04728] [EC:2.7.11.1] | |
| 572 | *BAD* | BCL2 associated agonist of cell death [KO:K02158] | |
| 578 | *BAK1* | BCL2 antagonist/killer 1 [KO:K14021] | |
| 581 | *BAX* | BCL2 associated X, apoptosis regulator [KO:K02159] | |
| 27113 | *BBC3* | BCL2 binding component 3 [KO:K10132] | |
| 596 | *BCL2* | BCL2, apoptosis regulator [KO:K02161] | |
| 597 | *BCL2A1* | BCL2 related protein A1 [KO:K02162] | |
| 598 | *BCL2L1* | BCL2 like 1 [KO:K04570] | |
| 10018 | *BCL2L11* | BCL2 like 11 [KO:K16341] | |
| 637 | *BID* | BH3 interacting domain death agonist [KO:K04726] | |
| 329 | *BIRC2* | baculoviral IAP repeat containing 2 [KO:K16060] | |
| 330 | *BIRC3* | baculoviral IAP repeat containing 3 [KO:K16060] | |
| 332 | *BIRC5* | baculoviral IAP repeat containing 5 [KO:K08731] | |
| 823 | *CAPN1* | calpain 1 [KO:K01367] [EC:3.4.22.52] | |
| 824 | *CAPN2* | calpain 2 [KO:K03853] [EC:3.4.22.53] | |
| 843 | *CASP10* | caspase 10 [KO:K04400] [EC:3.4.22.63] | |
| 1.01E+08 | *CASP12* | caspase 12 (gene/pseudogene) [KO:K04741] [EC:3.4.22.-] | |
| 835 | *CASP2* | caspase 2 [KO:K02186] [EC:3.4.22.55] | |
| 836 | *CASP3* | caspase 3 [KO:K02187] [EC:3.4.22.56] | |
| 839 | *CASP6* | caspase 6 [KO:K04396] [EC:3.4.22.59] | |
| 840 | *CASP7* | caspase 7 [KO:K04397] [EC:3.4.22.60] | |
| 841 | *CASP8* | caspase 8 [KO:K04398] [EC:3.4.22.61] | |
| 842 | *CASP9* | caspase 9 [KO:K04399] [EC:3.4.22.62] | |
| 8837 | *CFLAR* | CASP8 and FADD like apoptosis regulator [KO:K04724] | |
| 1147 | *CHUK* | conserved helix-loop-helix ubiquitous kinase [KO:K04467] [EC:2.7.11.10] | |
| 1439 | *CSF2RB* | colony stimulating factor 2 receptor beta common subunit [KO:K04738] | |
| 1508 | *CTSB* | cathepsin B [KO:K01363] [EC:3.4.22.1] | |
| 1075 | *CTSC* | cathepsin C [KO:K01275] [EC:3.4.14.1] | |
| 1509 | *CTSD* | cathepsin D [KO:K01379] [EC:3.4.23.5] | |
| 8722 | *CTSF* | cathepsin F [KO:K01373] [EC:3.4.22.41] | |
| 1512 | *CTSH* | cathepsin H [KO:K01366] [EC:3.4.22.16] | |
| 1513 | *CTSK* | cathepsin K [KO:K01371] [EC:3.4.22.38] | |
| 1514 | *CTSL* | cathepsin L [KO:K01365] [EC:3.4.22.15] | |
| 1519 | *CTSO* | cathepsin O [KO:K01374] [EC:3.4.22.42] | |
| 1520 | *CTSS* | cathepsin S [KO:K01368] [EC:3.4.22.27] | |
| 1515 | *CTSV* | cathepsin V [KO:K01375] [EC:3.4.22.43] | |
| 1521 | *CTSW* | cathepsin W [KO:K08569] [EC:3.4.22.-] | |
| 1522 | *CTSZ* | cathepsin Z [KO:K08568] [EC:3.4.18.1] | |
| 54205 | *CYCS* | cytochrome c, somatic [KO:K08738] | |
| 153090 | *DAB2IP* | DAB2 interacting protein [KO:K19901] | |
| 1616 | *DAXX* | death domain associated protein [KO:K02308] | |
| 1649 | *DDIT3* | DNA damage inducible transcript 3 [KO:K04452] | |
| 1676 | *DFFA* | DNA fragmentation factor subunit alpha [KO:K02310] | |
| 1677 | *DFFB* | DNA fragmentation factor subunit beta [KO:K02311] [EC:3.-.-.-] | |
| 56616 | *DIABLO* | diablo IAP-binding mitochondrial protein [KO:K10522] | |
| 9451 | *EIF2AK3* | eukaryotic translation initiation factor 2 alpha kinase 3 [KO:K08860] [EC:2.7.11.1] | |
| 1965 | *EIF2S1* | eukaryotic translation initiation factor 2 subunit alpha [KO:K03237] | |
| 2021 | *ENDOG* | endonuclease G [KO:K01173] | |
| 2081 | *ERN1* | endoplasmic reticulum to nucleus signaling 1 [KO:K08852] [EC:3.1.26.- 2.7.11.1] | |
| 8772 | *FADD* | Fas associated via death domain [KO:K02373] | |
| 355 | *FAS* | Fas cell surface death receptor [KO:K04390] | |
| 356 | *FASLG* | Fas ligand [KO:K04389] | |
| 2353 | *FOS* | Fos proto-oncogene, AP-1 transcription factor subunit [KO:K04379] | |
| 1647 | *GADD45A* | growth arrest and DNA damage inducible alpha [KO:K04402] | |
| 4616 | *GADD45B* | growth arrest and DNA damage inducible beta [KO:K04402] | |
| 10912 | *GADD45G* | growth arrest and DNA damage inducible gamma [KO:K04402] | |
| 3002 | *GZMB* | granzyme B [KO:K01353] [EC:3.4.21.79] | |
| 3265 | *HRAS* | HRas proto-oncogene, GTPase [KO:K02833] | |
| 8739 | *HRK* | harakiri, BCL2 interacting protein [KO:K02512] | |
| 27429 | *HTRA2* | HtrA serine peptidase 2 [KO:K08669] [EC:3.4.21.108] | |
| 3551 | *IKBKB* | inhibitor of nuclear factor kappa B kinase subunit beta [KO:K07209] [EC:2.7.11.10] | |
| 8517 | *IKBKG* | inhibitor of nuclear factor kappa B kinase subunit gamma [KO:K07210] | |
| 3562 | *IL3* | interleukin 3 [KO:K04736] | |
| 3563 | *IL3RA* | interleukin 3 receptor subunit alpha [KO:K04737] | |
| 3708 | *ITPR1* | inositol 1,4,5-trisphosphate receptor type 1 [KO:K04958] | |
| 3709 | *ITPR2* | inositol 1,4,5-trisphosphate receptor type 2 [KO:K04959] | |
| 3710 | *ITPR3* | inositol 1,4,5-trisphosphate receptor type 3 [KO:K04960] | |
| 3725 | *JUN* | Jun proto-oncogene, AP-1 transcription factor subunit [KO:K04448] | |
| 3845 | *KRAS* | KRAS proto-oncogene, GTPase [KO:K07827] | |
| 4000 | *LMNA* | lamin A/C [KO:K12641] | |
| 4001 | *LMNB1* | lamin B1 [KO:K07611] | |
| 84823 | *LMNB2* | lamin B2 [KO:K07611] | |
| 5604 | *MAP2K1* | mitogen-activated protein kinase kinase 1 [KO:K04368] [EC:2.7.12.2] | |
| 5605 | *MAP2K2* | mitogen-activated protein kinase kinase 2 [KO:K04369] [EC:2.7.12.2] | |
| 9020 | *MAP3K14* | mitogen-activated protein kinase kinase kinase 14 [KO:K04466] [EC:2.7.11.25] | |
| 4217 | *MAP3K5* | mitogen-activated protein kinase kinase kinase 5 [KO:K04426] [EC:2.7.11.25] | |
| 5594 | *MAPK1* | mitogen-activated protein kinase 1 [KO:K04371] [EC:2.7.11.24] | |
| 5602 | *MAPK10* | mitogen-activated protein kinase 10 [KO:K04440] [EC:2.7.11.24] | |
| 5595 | *MAPK3* | mitogen-activated protein kinase 3 [KO:K04371] [EC:2.7.11.24] | |
| 5599 | *MAPK8* | mitogen-activated protein kinase 8 [KO:K04440] [EC:2.7.11.24] | |
| 5601 | *MAPK9* | mitogen-activated protein kinase 9 [KO:K04440] [EC:2.7.11.24] | |
| 4170 | *MCL1* | MCL1, BCL2 family apoptosis regulator [KO:K02539] | |
| 4790 | *NFKB1* | nuclear factor kappa B subunit 1 [KO:K02580] | |
| 4792 | *NFKBIA* | NFKB inhibitor alpha [KO:K04734] | |
| 4803 | *NGF* | nerve growth factor [KO:K02582] | |
| 4893 | *NRAS* | NRAS proto-oncogene, GTPase [KO:K07828] | |
| 4914 | *NTRK1* | neurotrophic receptor tyrosine kinase 1 [KO:K03176] [EC:2.7.10.1] | |
| 142 | *PARP1* | poly(ADP-ribose) polymerase 1 [KO:K10798] [EC:2.4.2.30] | |
| 10038 | *PARP2* | poly(ADP-ribose) polymerase 2 [KO:K10798] [EC:2.4.2.30] | |
| 10039 | *PARP3* | poly(ADP-ribose) polymerase family member 3 [KO:K10798] [EC:2.4.2.30] | |
| 143 | *PARP4* | poly(ADP-ribose) polymerase family member 4 [KO:K10798] [EC:2.4.2.30] | |
| 5170 | *PDPK1* | 3-phosphoinositide dependent protein kinase 1 [KO:K06276] [EC:2.7.11.1] | |
| 55367 | *PIDD1* | p53-induced death domain protein 1 [KO:K10130] | |
| 5290 | *PIK3CA* | phosphatidylinositol-4,5-bisphosphate 3-kinase catalytic subunit alpha [KO:K00922] [EC:2.7.1.153] | |
| 5291 | *PIK3CB* | phosphatidylinositol-4,5-bisphosphate 3-kinase catalytic subunit beta [KO:K00922] [EC:2.7.1.153] | |
| 5293 | *PIK3CD* | phosphatidylinositol-4,5-bisphosphate 3-kinase catalytic subunit delta [KO:K00922] [EC:2.7.1.153] | |
| 5295 | *PIK3R1* | phosphoinositide-3-kinase regulatory subunit 1 [KO:K02649] | |
| 5296 | *PIK3R2* | phosphoinositide-3-kinase regulatory subunit 2 [KO:K02649] | |
| 8503 | *PIK3R3* | phosphoinositide-3-kinase regulatory subunit 3 [KO:K02649] | |
| 5366 | *PMAIP1* | phorbol-12-myristate-13-acetate-induced protein 1 [KO:K10131] | |
| 5551 | *PRF1* | perforin 1 [KO:K07818] | |
| 5783 | *PTPN13* | protein tyrosine phosphatase, non-receptor type 13 [KO:K02374] | |
| 5894 | *RAF1* | Raf-1 proto-oncogene, serine/threonine kinase [KO:K04366] [EC:2.7.11.1] | |
| 5970 | *RELA* | RELA proto-oncogene, NF-kB subunit [KO:K04735] | |
| 8737 | *RIPK1* | receptor interacting serine/threonine kinase 1 [KO:K02861] [EC:2.7.11.1] | |
| 5414 | *SEPT4* | septin 4 [KO:K16943] | |
| 6708 | *SPTA1* | spectrin alpha, erythrocytic 1 [KO:K06114] | |
| 6709 | *SPTAN1* | spectrin alpha, non-erythrocytic 1 [KO:K06114] | |
| 7124 | *TNF* | tumor necrosis factor [KO:K03156] | |
| 8797 | *TNFRSF10A* | TNF receptor superfamily member 10a [KO:K04722] | |
| 8795 | *TNFRSF10B* | TNF receptor superfamily member 10b [KO:K04722] | |
| 8794 | *TNFRSF10C* | TNF receptor superfamily member 10c [KO:K04722] | |
| 8793 | *TNFRSF10D* | TNF receptor superfamily member 10d [KO:K04722] | |
| 7132 | *TNFRSF1A* | TNF receptor superfamily member 1A [KO:K03158] | |
| 8743 | *TNFSF10* | TNF superfamily member 10 [KO:K04721] | |
| 7157 | *TP53* | tumor protein p53 [KO:K04451] | |
| 63970 | *TP53AIP1* | tumor protein p53 regulated apoptosis inducing protein 1 [KO:K13773] | |
| 8717 | *TRADD* | TNFRSF1A associated via death domain [KO:K03171] | |
| 7185 | *TRAF1* | TNF receptor associated factor 1 [KO:K03172] | |
| 7186 | *TRAF2* | TNF receptor associated factor 2 [KO:K03173] [EC:2.3.2.27] | |
| 7846 | *TUBA1A* | tubulin alpha 1a [KO:K07374] | |
| 10376 | *TUBA1B* | tubulin alpha 1b [KO:K07374] | |
| 84790 | *TUBA1C* | tubulin alpha 1c [KO:K07374] | |
| 7278 | *TUBA3C* | tubulin alpha 3c [KO:K07374] | |
| 113457 | *TUBA3D* | tubulin alpha 3d [KO:K07374] | |
| 112714 | *TUBA3E* | tubulin alpha 3e [KO:K07374] | |
| 7277 | *TUBA4A* | tubulin alpha 4a [KO:K07374] | |
| 51807 | *TUBA8* | tubulin alpha 8 [KO:K07374] | |
| 79861 | *TUBAL3* | tubulin alpha like 3 [KO:K07374] | |
| 331 | *XIAP* | X-linked inhibitor of apoptosis [KO:K04725] [EC:2.3.2.27] | |

| Supplemental Table 2. Differential expression in KEGG Apoptosis Pathway in MSS tumors | | | | | | |
| --- | --- | --- | --- | --- | --- | --- |
| *Gene Name* | Tumor Mean | Normal Mean | Fold Change | P-Value | | Adjusted P-Value |
| *MAPK10* | 9.11 | 27.60 | 0.33 | 1.54E-31 | 2.27E-30 | |
| *SPTA1* | 0.70 | 2.02 | 0.35 | 4.35E-05 | 7.32E-05 | |
| *CSF2RB* | 26.28 | 73.36 | 0.36 | 3.01E-35 | 1.00E-33 | |
| *BCL2* | 22.74 | 61.38 | 0.37 | 1.93E-36 | 8.56E-35 | |
| *TUBAL3* | 3.10 | 8.08 | 0.38 | 2.53E-13 | 7.16E-13 | |
| *NTRK1* | 0.95 | 2.40 | 0.39 | 2.24E-08 | 4.45E-08 | |
| *FOS* | 185.07 | 459.66 | 0.40 | 2.51E-27 | 2.57E-26 | |
| *CASP12* | 0.41 | 0.92 | 0.45 | 1.40E-03 | 1.98E-03 | |
| *FAS* | 25.99 | 49.72 | 0.52 | 3.54E-23 | 2.62E-22 | |
| *ITPR1* | 57.25 | 109.06 | 0.52 | 1.72E-31 | 2.28E-30 | |
| *FASLG* | 0.91 | 1.69 | 0.54 | 3.27E-04 | 5.05E-04 | |
| *CASP10* | 62.68 | 113.40 | 0.55 | 8.86E-35 | 2.36E-33 | |
| *TUBA8* | 3.05 | 5.35 | 0.57 | 9.18E-09 | 1.94E-08 | |
| *PIK3CD* | 26.55 | 45.40 | 0.58 | 3.46E-19 | 1.39E-18 | |
| *LMNA* | 235.76 | 395.20 | 0.60 | 3.52E-22 | 2.13E-21 | |
| *BCL2L11* | 53.91 | 87.47 | 0.62 | 8.31E-23 | 5.26E-22 | |
| *CTSS* | 114.71 | 183.60 | 0.62 | 3.19E-21 | 1.65E-20 | |
| *CASP7* | 57.94 | 92.59 | 0.63 | 2.46E-20 | 1.13E-19 | |
| *BIRC3* | 71.93 | 113.35 | 0.63 | 7.98E-15 | 2.47E-14 | |
| *TNFSF10* | 45.06 | 70.25 | 0.64 | 1.21E-13 | 3.49E-13 | |
| *EIF2AK3* | 50.69 | 78.83 | 0.64 | 4.68E-23 | 3.27E-22 | |
| *IL3RA* | 2.47 | 3.82 | 0.65 | 6.79E-03 | 8.94E-03 | |
| *MAP3K14* | 33.49 | 51.65 | 0.65 | 1.30E-23 | 1.02E-22 | |
| *MAPK3* | 62.23 | 93.18 | 0.67 | 4.28E-17 | 1.62E-16 | |
| *TP53AIP1* | 0.52 | 0.76 | 0.69 | 8.91E-02 | 1.06E-01 | |
| *TUBA3E* | 0.46 | 0.67 | 0.69 | 4.47E-02 | 5.71E-02 | |
| *CFLAR* | 202.93 | 293.76 | 0.69 | 6.56E-28 | 7.27E-27 | |
| *CTSW* | 2.68 | 3.86 | 0.69 | 6.26E-04 | 9.35E-04 | |
| *BAD* | 13.02 | 18.65 | 0.70 | 5.04E-12 | 1.24E-11 | |
| *CAPN2* | 239.62 | 342.95 | 0.70 | 7.83E-22 | 4.53E-21 | |
| *RIPK1* | 49.00 | 69.33 | 0.71 | 1.71E-16 | 5.83E-16 | |
| *ERN1* | 27.61 | 38.79 | 0.71 | 2.54E-09 | 5.62E-09 | |
| *CASP9* | 12.91 | 17.67 | 0.73 | 3.19E-07 | 5.90E-07 | |
| *TNFRSF1A* | 87.61 | 118.88 | 0.74 | 7.39E-23 | 4.91E-22 | |
| *NFKBIA* | 58.25 | 76.46 | 0.76 | 5.31E-08 | 1.04E-07 | |
| *ATM* | 234.61 | 305.26 | 0.77 | 7.79E-14 | 2.35E-13 | |
| *MCL1* | 605.99 | 787.79 | 0.77 | 1.43E-12 | 3.66E-12 | |
| *CYCS* | 94.29 | 122.00 | 0.77 | 8.10E-10 | 1.86E-09 | |
| *DAB2IP* | 146.23 | 188.74 | 0.77 | 1.41E-12 | 3.66E-12 | |
| *AKT3* | 40.61 | 52.05 | 0.78 | 5.75E-05 | 9.56E-05 | |
| *BAK1* | 19.58 | 24.25 | 0.81 | 8.81E-06 | 1.54E-05 | |
| *BIRC2* | 78.61 | 95.62 | 0.82 | 1.04E-09 | 2.35E-09 | |
| *CTSD* | 275.51 | 331.09 | 0.83 | 3.91E-06 | 6.93E-06 | |
| *ITPR2* | 219.48 | 258.14 | 0.85 | 1.45E-03 | 2.03E-03 | |
| *CTSF* | 7.70 | 9.05 | 0.85 | 8.33E-02 | 1.01E-01 | |
| *TRADD* | 25.47 | 29.48 | 0.86 | 4.22E-03 | 5.66E-03 | |
| *RAF1* | 129.93 | 147.72 | 0.88 | 1.88E-07 | 3.53E-07 | |
| *MAP3K5* | 64.53 | 73.00 | 0.88 | 9.98E-04 | 1.44E-03 | |
| *TNFRSF10D* | 20.02 | 22.64 | 0.88 | 5.58E-02 | 7.00E-02 | |
| *KRAS* | 102.76 | 115.27 | 0.89 | 6.55E-03 | 8.71E-03 | |
| *NFKB1* | 74.35 | 83.07 | 0.90 | 5.04E-04 | 7.70E-04 | |
| *GADD45G* | 2.84 | 3.09 | 0.92 | 4.65E-01 | 4.90E-01 | |
| *TRAF1* | 46.94 | 50.77 | 0.92 | 1.02E-01 | 1.20E-01 | |
| *CASP3* | 40.44 | 43.73 | 0.92 | 1.24E-01 | 1.43E-01 | |
| *PARP2* | 19.64 | 21.19 | 0.93 | 2.00E-01 | 2.27E-01 | |
| *APAF1* | 84.80 | 91.19 | 0.93 | 5.17E-02 | 6.55E-02 | |
| *PARP3* | 18.38 | 19.76 | 0.93 | 2.20E-01 | 2.46E-01 | |
| *MAP2K2* | 71.00 | 76.18 | 0.93 | 4.26E-02 | 5.50E-02 | |
| *PIDD* | 29.34 | 31.18 | 0.94 | 2.03E-01 | 2.29E-01 | |
| *CTSC* | 80.75 | 84.83 | 0.95 | 2.38E-01 | 2.64E-01 | |
| *IKBKB* | 139.63 | 146.41 | 0.95 | 1.80E-01 | 2.07E-01 | |
| *PRF1* | 4.73 | 4.90 | 0.96 | 7.37E-01 | 7.48E-01 | |
| *SEPT4* | 5.88 | 6.09 | 0.97 | 7.00E-01 | 7.16E-01 | |
| *TNF* | 1.94 | 2.00 | 0.97 | 8.43E-01 | 8.43E-01 | |
| *AKT1* | 149.80 | 153.53 | 0.98 | 3.57E-01 | 3.83E-01 | |
| *MAPK8* | 60.07 | 59.48 | 1.01 | 7.92E-01 | 7.98E-01 | |
| *MAPK1* | 179.19 | 177.32 | 1.01 | 6.62E-01 | 6.88E-01 | |
| *PIK3R3* | 33.81 | 32.81 | 1.03 | 5.75E-01 | 6.02E-01 | |
| *MAPK9* | 64.79 | 62.72 | 1.03 | 3.81E-01 | 4.06E-01 | |
| *PIK3R1* | 152.79 | 146.65 | 1.04 | 2.54E-01 | 2.79E-01 | |
| *TUBA3D* | 2.85 | 2.73 | 1.04 | 6.83E-01 | 7.04E-01 | |
| *JUN* | 229.02 | 218.97 | 1.05 | 3.18E-01 | 3.44E-01 | |
| *CASP6* | 22.59 | 20.66 | 1.09 | 8.54E-02 | 1.02E-01 | |
| *PDPK1* | 105.88 | 96.01 | 1.10 | 1.70E-03 | 2.35E-03 | |
| *IKBKG* | 7.43 | 6.74 | 1.10 | 2.64E-01 | 2.88E-01 | |
| *CTSL1* | 18.70 | 16.85 | 1.11 | 1.09E-01 | 1.27E-01 | |
| *ITPR3* | 422.46 | 378.16 | 1.12 | 1.77E-04 | 2.84E-04 | |
| *PIK3CB* | 90.41 | 80.84 | 1.12 | 1.33E-03 | 1.91E-03 | |
| *RELA* | 83.08 | 74.02 | 1.12 | 1.40E-05 | 2.42E-05 | |
| *DIABLO* | 39.22 | 34.91 | 1.12 | 5.55E-04 | 8.39E-04 | |
| *ENDOG* | 12.61 | 11.04 | 1.14 | 4.23E-02 | 5.50E-02 | |
| *GADD45A* | 12.80 | 11.20 | 1.14 | 6.08E-02 | 7.56E-02 | |
| *GADD45B* | 13.22 | 11.56 | 1.14 | 7.81E-02 | 9.53E-02 | |
| *MAP2K1* | 31.83 | 27.72 | 1.15 | 3.42E-03 | 4.69E-03 | |
| *SPTAN1* | 542.83 | 472.74 | 1.15 | 6.26E-08 | 1.21E-07 | |
| *CAPN1* | 190.07 | 164.47 | 1.16 | 8.15E-08 | 1.55E-07 | |
| *CHUK* | 42.41 | 36.69 | 1.16 | 4.17E-03 | 5.66E-03 | |
| *AKT2* | 159.97 | 136.80 | 1.17 | 3.68E-07 | 6.70E-07 | |
| *CTSZ* | 128.19 | 109.35 | 1.17 | 7.40E-05 | 1.21E-04 | |
| *HTRA2* | 19.47 | 16.07 | 1.21 | 3.69E-05 | 6.28E-05 | |
| *DFFB* | 16.13 | 13.21 | 1.22 | 8.50E-04 | 1.24E-03 | |
| *XIAP* | 190.20 | 155.47 | 1.22 | 8.83E-13 | 2.40E-12 | |
| *ATF4* | 128.67 | 104.89 | 1.23 | 3.56E-11 | 8.45E-11 | |
| *CASP8* | 68.98 | 56.19 | 1.23 | 9.31E-09 | 1.94E-08 | |
| *FADD* | 14.56 | 11.77 | 1.24 | 7.81E-04 | 1.15E-03 | |
| *BAX* | 36.92 | 29.76 | 1.24 | 1.80E-04 | 2.85E-04 | |
| *PIK3CA* | 67.41 | 53.01 | 1.27 | 1.40E-08 | 2.85E-08 | |
| *BID* | 35.38 | 27.51 | 1.29 | 1.55E-08 | 3.13E-08 | |
| *TNFRSF10A* | 36.66 | 28.31 | 1.30 | 1.63E-06 | 2.93E-06 | |
| *DAXX* | 35.84 | 27.39 | 1.31 | 4.69E-11 | 1.09E-10 | |
| *DDIT3* | 10.78 | 8.23 | 1.31 | 2.46E-04 | 3.86E-04 | |
| *PIK3R2* | 73.15 | 54.17 | 1.35 | 5.20E-17 | 1.92E-16 | |
| *PARP1* | 105.04 | 77.19 | 1.36 | 8.30E-14 | 2.45E-13 | |
| *ACTB* | 2149.33 | 1571.97 | 1.37 | 6.05E-27 | 5.75E-26 | |
| *BCL2A1* | 1.80 | 1.31 | 1.37 | 6.63E-02 | 8.16E-02 | |
| *EIF2S1* | 67.05 | 48.74 | 1.38 | 3.39E-13 | 9.39E-13 | |
| *TRAF2* | 40.71 | 29.42 | 1.38 | 6.02E-12 | 1.46E-11 | |
| *DFFA* | 84.80 | 60.44 | 1.40 | 1.26E-20 | 6.00E-20 | |
| *NRAS* | 105.16 | 74.25 | 1.42 | 1.73E-15 | 5.48E-15 | |
| *CTSB* | 498.59 | 351.13 | 1.42 | 2.72E-19 | 1.13E-18 | |
| *CASP2* | 121.60 | 85.38 | 1.42 | 1.03E-20 | 5.08E-20 | |
| *HRAS* | 17.71 | 12.32 | 1.44 | 7.06E-09 | 1.52E-08 | |
| *ACTG1* | 1194.58 | 821.69 | 1.45 | 1.18E-24 | 9.85E-24 | |
| *TUBA1A* | 30.71 | 20.86 | 1.47 | 3.10E-09 | 6.76E-09 | |
| *TNFRSF10B* | 133.04 | 90.09 | 1.48 | 8.95E-17 | 3.22E-16 | |
| *PARP4* | 359.90 | 226.86 | 1.59 | 9.56E-27 | 8.48E-26 | |
| *AIFM1* | 42.45 | 26.69 | 1.59 | 2.71E-18 | 1.06E-17 | |
| *CTSH* | 79.60 | 49.98 | 1.59 | 6.07E-16 | 1.97E-15 | |
| *CTSK* | 49.47 | 30.40 | 1.63 | 3.33E-12 | 8.36E-12 | |
| *TNFRSF10C* | 3.51 | 2.09 | 1.68 | 1.36E-04 | 2.21E-04 | |
| *TUBA4A* | 33.61 | 19.56 | 1.72 | 3.23E-21 | 1.65E-20 | |
| *TP53* | 99.81 | 57.76 | 1.73 | 1.16E-19 | 5.16E-19 | |
| *TUBA1B* | 134.48 | 76.55 | 1.76 | 5.39E-32 | 8.97E-31 | |
| *BBC3* | 20.96 | 11.48 | 1.82 | 1.01E-16 | 3.53E-16 | |
| *LMNB1* | 83.22 | 41.86 | 1.99 | 9.27E-33 | 1.76E-31 | |
| *TUBA1C* | 71.67 | 34.46 | 2.08 | 8.71E-30 | 1.05E-28 | |
| *LMNB2* | 146.17 | 68.56 | 2.13 | 9.15E-41 | 6.08E-39 | |
| *PTPN13* | 68.24 | 31.41 | 2.17 | 2.69E-19 | 1.13E-18 | |
| *BCL2L1* | 151.06 | 63.73 | 2.37 | 4.40E-54 | 5.85E-52 | |
| *BIRC5* | 34.06 | 11.20 | 3.04 | 7.93E-33 | 1.76E-31 | |
| *PMAIP1* | 9.77 | 3.16 | 3.10 | 2.06E-21 | 1.14E-20 | |
| *GZMB* | 4.16 | 1.22 | 3.41 | 1.30E-12 | 3.45E-12 | |
| *CTSL2* | 4.64 | 1.17 | 3.97 | 2.63E-16 | 8.75E-16 | |

| Supplemental Table 3. Differentially expressed genes in KEGG Apoptosis Pathway in MSI tumors | | | | | |
| --- | --- | --- | --- | --- | --- |
| Gene Name | Tumor Mean | Normal Mean | Fold Change | P-Value | Adjusted P-Value |
| *SPTA1* | 1.25 | 5.92 | 0.21 | 1.25E-02 | 3.61E-02 |
| *TUBAL3* | 2.55 | 9.66 | 0.26 | 5.21E-04 | 3.01E-03 |
| *TUBA3E* | 0.34 | 1.21 | 0.28 | 9.17E-02 | 1.79E-01 |
| *IL3RA* | 0.32 | 1.04 | 0.31 | 1.84E-01 | 3.01E-01 |
| *MAPK10* | 10.30 | 32.15 | 0.32 | 3.94E-04 | 2.38E-03 |
| *CTSF* | 3.74 | 8.42 | 0.44 | 1.51E-03 | 7.17E-03 |
| *CASP12* | 0.50 | 1.07 | 0.47 | 3.35E-01 | 4.73E-01 |
| *ITPR1* | 56.72 | 115.88 | 0.49 | 5.30E-06 | 1.41E-04 |
| *CSF2RB* | 38.29 | 76.14 | 0.50 | 5.66E-05 | 5.79E-04 |
| *FOS* | 205.26 | 406.32 | 0.51 | 1.13E-03 | 5.57E-03 |
| *BCL2* | 36.13 | 68.16 | 0.53 | 2.78E-05 | 3.36E-04 |
| *TUBA3D* | 3.23 | 5.41 | 0.60 | 9.52E-02 | 1.80E-01 |
| *CASP7* | 75.03 | 125.52 | 0.60 | 9.40E-06 | 1.65E-04 |
| *PIK3CD* | 33.57 | 54.97 | 0.61 | 1.05E-03 | 5.39E-03 |
| *GADD45G* | 2.45 | 3.90 | 0.63 | 1.09E-01 | 1.97E-01 |
| *CTSS* | 108.89 | 159.27 | 0.68 | 3.08E-03 | 1.36E-02 |
| *MAP3K14* | 35.95 | 51.93 | 0.69 | 4.17E-03 | 1.60E-02 |
| *ERN1* | 24.05 | 34.47 | 0.70 | 3.01E-03 | 1.36E-02 |
| *GADD45B* | 10.44 | 14.69 | 0.71 | 9.38E-02 | 1.80E-01 |
| *MAPK3* | 64.20 | 89.11 | 0.72 | 1.31E-02 | 3.70E-02 |
| *RIPK1* | 52.25 | 72.08 | 0.72 | 2.34E-04 | 1.63E-03 |
| *ATM* | 269.39 | 371.32 | 0.73 | 6.90E-04 | 3.82E-03 |
| *FAS* | 44.41 | 61.21 | 0.73 | 3.67E-03 | 1.52E-02 |
| *TUBA8* | 5.16 | 6.85 | 0.75 | 9.81E-02 | 1.81E-01 |
| *TRAF1* | 45.11 | 59.84 | 0.75 | 3.86E-02 | 9.01E-02 |
| *BCL2L11* | 74.18 | 97.99 | 0.76 | 2.19E-02 | 5.60E-02 |
| *TNF* | 2.01 | 2.65 | 0.76 | 4.33E-01 | 5.50E-01 |
| *CFLAR* | 210.11 | 271.49 | 0.77 | 5.20E-03 | 1.92E-02 |
| *DAB2IP* | 132.96 | 168.96 | 0.79 | 8.04E-03 | 2.79E-02 |
| *CASP9* | 14.42 | 18.12 | 0.80 | 1.89E-02 | 5.14E-02 |
| *JUN* | 132.30 | 166.08 | 0.80 | 6.57E-02 | 1.37E-01 |
| *AKT3* | 44.94 | 56.10 | 0.80 | 1.11E-01 | 1.97E-01 |
| *CTSZ* | 89.18 | 109.86 | 0.81 | 4.57E-02 | 1.03E-01 |
| *CYCS* | 101.28 | 124.16 | 0.82 | 5.30E-02 | 1.17E-01 |
| *MCL1* | 687.13 | 841.34 | 0.82 | 1.12E-02 | 3.47E-02 |
| *NTRK1* | 3.23 | 3.88 | 0.83 | 6.69E-01 | 7.48E-01 |
| *BIRC3* | 153.28 | 183.89 | 0.83 | 2.05E-01 | 3.24E-01 |
| *IKBKB* | 120.32 | 143.21 | 0.84 | 2.52E-02 | 6.32E-02 |
| *CASP10* | 85.05 | 101.10 | 0.84 | 2.24E-01 | 3.47E-01 |
| *CAPN2* | 274.42 | 324.61 | 0.85 | 3.05E-02 | 7.38E-02 |
| *EIF2AK3* | 61.01 | 72.16 | 0.85 | 7.04E-02 | 1.42E-01 |
| *CTSD* | 243.03 | 282.26 | 0.86 | 7.22E-02 | 1.43E-01 |
| *PIDD* | 26.78 | 30.88 | 0.87 | 3.12E-01 | 4.51E-01 |
| *SEPT4* | 7.46 | 8.58 | 0.87 | 5.37E-01 | 6.27E-01 |
| *CTSW* | 3.52 | 4.04 | 0.87 | 4.38E-01 | 5.50E-01 |
| *MAP2K2* | 64.51 | 73.95 | 0.87 | 1.65E-01 | 2.74E-01 |
| *BAD* | 14.24 | 16.27 | 0.88 | 4.28E-01 | 5.50E-01 |
| *TRADD* | 22.94 | 25.98 | 0.88 | 2.83E-01 | 4.28E-01 |
| *CASP6* | 22.15 | 24.80 | 0.89 | 3.34E-01 | 4.73E-01 |
| *BIRC2* | 106.17 | 117.87 | 0.90 | 1.24E-01 | 2.13E-01 |
| *ITPR2* | 213.13 | 235.90 | 0.90 | 4.18E-01 | 5.50E-01 |
| *CTSH* | 59.04 | 64.98 | 0.91 | 3.68E-01 | 5.05E-01 |
| *PIK3CA* | 63.20 | 69.17 | 0.91 | 3.01E-01 | 4.45E-01 |
| *TNFSF10* | 72.80 | 79.33 | 0.92 | 4.66E-01 | 5.74E-01 |
| *PDPK1* | 71.62 | 77.35 | 0.93 | 1.92E-01 | 3.11E-01 |
| *NFKBIA* | 63.25 | 66.20 | 0.96 | 7.00E-01 | 7.76E-01 |
| *TNFRSF1A* | 111.85 | 116.86 | 0.96 | 5.80E-01 | 6.70E-01 |
| *PARP3* | 22.07 | 22.84 | 0.97 | 7.58E-01 | 8.33E-01 |
| *BCL2A1* | 3.87 | 3.99 | 0.97 | 9.29E-01 | 9.52E-01 |
| *LMNA* | 314.87 | 320.54 | 0.98 | 8.79E-01 | 9.35E-01 |
| *PIK3R1* | 164.89 | 166.17 | 0.99 | 9.38E-01 | 9.52E-01 |
| *RAF1* | 143.83 | 143.94 | 1.00 | 9.89E-01 | 9.89E-01 |
| *PARP4* | 219.72 | 218.56 | 1.01 | 9.01E-01 | 9.43E-01 |
| *CTSC* | 107.40 | 106.80 | 1.01 | 9.46E-01 | 9.53E-01 |
| *MAPK9* | 74.49 | 74.05 | 1.01 | 9.36E-01 | 9.52E-01 |
| *APAF1* | 89.61 | 88.41 | 1.01 | 8.60E-01 | 9.22E-01 |
| *BAK1* | 23.65 | 23.24 | 1.02 | 8.92E-01 | 9.41E-01 |
| *TUBA1A* | 13.88 | 13.38 | 1.04 | 8.03E-01 | 8.75E-01 |
| *TP53AIP1* | 2.00 | 1.93 | 1.04 | 9.33E-01 | 9.52E-01 |
| *XIAP* | 167.14 | 160.50 | 1.04 | 4.80E-01 | 5.86E-01 |
| *PRF1* | 8.76 | 8.38 | 1.05 | 8.25E-01 | 8.93E-01 |
| *DAXX* | 34.38 | 32.89 | 1.05 | 6.55E-01 | 7.38E-01 |
| *AKT2* | 136.94 | 130.43 | 1.05 | 4.35E-01 | 5.50E-01 |
| *BAX* | 31.25 | 29.59 | 1.06 | 6.42E-01 | 7.30E-01 |
| *CAPN1* | 174.64 | 165.06 | 1.06 | 4.59E-01 | 5.71E-01 |
| *MAPK8* | 75.77 | 71.01 | 1.07 | 3.89E-01 | 5.28E-01 |
| *AIFM1* | 32.25 | 30.10 | 1.07 | 6.29E-01 | 7.21E-01 |
| *KRAS* | 142.77 | 132.38 | 1.08 | 3.09E-01 | 4.51E-01 |
| *NRAS* | 100.87 | 93.26 | 1.08 | 2.99E-01 | 4.45E-01 |
| *HTRA2* | 18.16 | 16.52 | 1.10 | 4.90E-01 | 5.87E-01 |
| *MAPK1* | 215.16 | 195.12 | 1.10 | 6.55E-02 | 1.37E-01 |
| *PARP2* | 26.03 | 23.41 | 1.11 | 3.57E-01 | 4.95E-01 |
| *PIK3CB* | 94.60 | 84.83 | 1.12 | 1.95E-01 | 3.12E-01 |
| *RELA* | 77.61 | 69.46 | 1.12 | 1.64E-01 | 2.74E-01 |
| *FADD* | 15.43 | 13.79 | 1.12 | 4.34E-01 | 5.50E-01 |
| *NFKB1* | 101.45 | 90.62 | 1.12 | 1.14E-01 | 1.99E-01 |
| *CTSK* | 40.32 | 35.97 | 1.12 | 5.35E-01 | 6.27E-01 |
| *CASP8* | 81.67 | 72.25 | 1.13 | 2.14E-01 | 3.35E-01 |
| *GADD45A* | 14.62 | 12.89 | 1.13 | 4.07E-01 | 5.47E-01 |
| *ENDOG* | 13.26 | 11.47 | 1.16 | 3.42E-01 | 4.79E-01 |
| *CASP3* | 61.68 | 52.95 | 1.16 | 1.25E-01 | 2.13E-01 |
| *ITPR3* | 421.50 | 359.17 | 1.17 | 4.36E-02 | 1.00E-01 |
| *ACTB* | 1969.14 | 1664.26 | 1.18 | 1.16E-02 | 3.51E-02 |
| *DIABLO* | 43.86 | 37.01 | 1.19 | 6.84E-02 | 1.40E-01 |
| *BID* | 37.98 | 30.85 | 1.23 | 6.06E-02 | 1.32E-01 |
| *IKBKG* | 4.38 | 3.53 | 1.24 | 5.30E-01 | 6.27E-01 |
| *TRAF2* | 37.45 | 30.19 | 1.24 | 6.14E-02 | 1.32E-01 |
| *PIK3R2* | 72.23 | 57.64 | 1.25 | 4.19E-03 | 1.60E-02 |
| *AKT1* | 181.80 | 143.79 | 1.26 | 3.16E-03 | 1.36E-02 |
| *DFFB* | 15.33 | 12.06 | 1.27 | 3.22E-02 | 7.66E-02 |
| *SPTAN1* | 548.26 | 430.34 | 1.27 | 3.94E-04 | 2.38E-03 |
| *MAP3K5* | 96.59 | 75.74 | 1.28 | 3.05E-02 | 7.38E-02 |
| *PIK3R3* | 54.55 | 42.40 | 1.29 | 9.61E-02 | 1.80E-01 |
| *CHUK* | 58.66 | 45.28 | 1.30 | 8.65E-03 | 2.88E-02 |
| *MAP2K1* | 39.05 | 30.06 | 1.30 | 1.99E-02 | 5.29E-02 |
| *ATF4* | 146.18 | 111.12 | 1.32 | 8.17E-03 | 2.79E-02 |
| *GZMB* | 5.20 | 3.82 | 1.36 | 4.25E-01 | 5.50E-01 |
| *FASLG* | 2.80 | 2.04 | 1.37 | 4.85E-01 | 5.86E-01 |
| *CASP2* | 144.95 | 102.97 | 1.41 | 3.32E-05 | 3.68E-04 |
| *CTSB* | 609.50 | 421.18 | 1.45 | 1.72E-04 | 1.27E-03 |
| *EIF2S1* | 90.21 | 62.00 | 1.46 | 2.88E-04 | 1.91E-03 |
| *DFFA* | 97.68 | 66.72 | 1.46 | 1.00E-04 | 7.86E-04 |
| *PARP1* | 145.09 | 98.80 | 1.47 | 1.01E-03 | 5.38E-03 |
| *TNFRSF10A* | 46.35 | 31.09 | 1.49 | 4.20E-03 | 1.60E-02 |
| *BCL2L1* | 98.91 | 65.91 | 1.50 | 8.61E-05 | 7.16E-04 |
| *TNFRSF10D* | 29.44 | 19.58 | 1.50 | 9.69E-03 | 3.14E-02 |
| *DDIT3* | 12.31 | 8.04 | 1.53 | 2.09E-02 | 5.44E-02 |
| *BBC3* | 18.07 | 11.70 | 1.54 | 1.22E-02 | 3.61E-02 |
| *ACTG1* | 1282.52 | 829.36 | 1.55 | 1.23E-05 | 1.81E-04 |
| *TUBA4A* | 32.30 | 20.62 | 1.57 | 6.35E-03 | 2.28E-02 |
| *CTSL1* | 29.48 | 18.54 | 1.59 | 1.08E-02 | 3.43E-02 |
| *TNFRSF10C* | 3.46 | 2.13 | 1.63 | 2.59E-01 | 3.97E-01 |
| *TNFRSF10B* | 145.93 | 86.81 | 1.68 | 9.93E-06 | 1.65E-04 |
| *PTPN13* | 83.56 | 44.48 | 1.88 | 1.76E-02 | 4.88E-02 |
| *TP53* | 138.21 | 72.39 | 1.91 | 7.60E-07 | 3.37E-05 |
| *HRAS* | 24.90 | 12.98 | 1.92 | 6.66E-06 | 1.48E-04 |
| *CTSL2* | 2.76 | 1.43 | 1.92 | 1.10E-01 | 1.97E-01 |
| *TUBA1B* | 151.33 | 69.89 | 2.17 | 6.31E-05 | 5.86E-04 |
| *TUBA1C* | 57.15 | 26.02 | 2.20 | 2.24E-05 | 2.98E-04 |
| *LMNB1* | 134.72 | 59.02 | 2.28 | 1.11E-06 | 3.68E-05 |
| *LMNB2* | 181.79 | 72.06 | 2.52 | 4.51E-07 | 3.00E-05 |
| *PMAIP1* | 21.89 | 5.84 | 3.75 | 6.61E-05 | 5.86E-04 |
| *BIRC5* | 46.64 | 12.07 | 3.86 | 3.66E-08 | 4.87E-06 |
